# Effect of Resting-State fNIRS Scanning Duration on Functional Brain Connectivity and Graph Theory Metrics of Brain Network

**DOI:** 10.3389/fnins.2017.00392

**Published:** 2017-07-20

**Authors:** Shujie Geng, Xiangyu Liu, Bharat B. Biswal, Haijing Niu

**Affiliations:** ^1^State Key Laboratory of Cognitive Neuroscience and Learning and IDG/McGovern Institute for Brain Research, Beijing Normal University Beijing, China; ^2^Center for Collaboration and Innovation in Brain and Learning Sciences, Beijing Normal University Beijing, China; ^3^Department of Neurology, Shenzhen Longhua District Central Hospital Guang dong, China; ^4^Department of Biomedical Engineering, New Jersey Institute of Technology, University Height Newark, NJ, United States; ^5^Beijing Key Laboratory of Brain Imaging and Connectomics, Beijing Normal University Beijing, China

**Keywords:** resting state, connectome, functional connectivity, graph, scanning duration, fNIRS

## Abstract

As an emerging brain imaging technique, functional near infrared spectroscopy (fNIRS) has attracted widespread attention for advancing resting-state functional connectivity (FC) and graph theoretical analyses of brain networks. However, it remains largely unknown how the duration of the fNIRS signal scanning is related to stable and reproducible functional brain network features. To answer this question, we collected resting-state fNIRS signals (10-min duration, two runs) from 18 participants and then truncated the hemodynamic time series into 30-s time bins that ranged from 1 to 10 min. Measures of nodal efficiency, nodal betweenness, network local efficiency, global efficiency, and clustering coefficient were computed for each subject at each fNIRS signal acquisition duration. Analyses of the stability and between-run reproducibility were performed to identify optimal time length for each measure. We found that the FC, nodal efficiency and nodal betweenness stabilized and were reproducible after 1 min of fNIRS signal acquisition, whereas network clustering coefficient, local and global efficiencies stabilized after 1 min and were reproducible after 5 min of fNIRS signal acquisition for only local and global efficiencies. These quantitative results provide direct evidence regarding the choice of the resting-state fNIRS scanning duration for functional brain connectivity and topological metric stability of brain network connectivity.

## Introduction

As an emerging brain imaging technique, functional near infrared spectroscopy (fNIRS) is attracting increasing interests for studying human brain functional organization. The fNIRS technique possesses several unique advantages compared to functional magnetic resonance imaging (fMRI), such as simultaneous recording of signal changes in both oxygenated and deoxygenated hemoglobin concentration, higher temporal resolution, and better portability for use (Niu and He, 2013).

Recent advances allow fNIRS to acquire whole-brain resting-state signals and to construct entire cortical functional brain networks. Using modern graph theoretical approaches, fNIRS-derived brain networks can be further quantified to obtain topological characteristics representing network organization configurations within the brain. Based on healthy adult data, our previous study revealed several important topological organizational principles from fNIRS brain networks, such as small-world property, modular structure, and highly connected hubs (Niu et al., 2012). The reproducibility and reliability of these network measures were also further validated based on our two-scanning-run resting-state data (Niu et al., 2013). In addition, in Fekete et al.'s study, the authors have also noted that the small-world properties of the prefrontal network derived from fNIRS-based data are associated with variability in young children's risk of developmental psychopathology (Fekete et al., 2014). To extend these studies to much wider applications, such as brain development and disease-associated studies, it is important for fNIRS data to be able to identify development/disease-associated changes in brain connectivity and topological metrics. Such changes may reflect functional markers of development/disease that could advance our understanding into brain nervous system function/dysfunction in the future.

It is generally necessary to perform several preprocessing procedures before constructing functional brain networks and computing graph theory metrics. These include collecting resting-state fNIRS time course data, preprocessing, estimating the correlation coefficient matrix, and analyzing the functional network using the graph theoretical method. Resting-state fNIRS data are typically collected for ~7–10 min (Niu and He, 2013). However, the scanning length required to collect fNIRS data would be challenging for brain development studies associated with infants and young children. Certainly, such long scanning duration could also be problematic for constrained clinical patients, particularly for clinical imaging protocols that include additional task-related experimental designs. Previous fMRI–derived brain imaging studies have suggested that 5~7 min (Van Dijk et al., 2010; Tomasi et al., 2016), or ≥9 min (Birn et al., 2013; Dawson et al., 2013; Laumann et al., 2015) BOLD data can yield stable correlation strengths and ~2 min BOLD data can yield stable graph theoretical metrics (Whitlow et al., 2011). However, the length of time in which the resting-state fNIRS imaging data duration can generate stable, test-retest reproducible functional connection, and graph theory metrics of brain network connectivity remains unknown. Such conclusions would provide important information for human brain development and for the clinical implementation of fNIRS-based techniques.

In the present study, functional brain network connectivity and graph theoretical analyses were applied to a series of incrementally longer temporal epochs of resting-state fNIRS imaging data. We hypothesized that functional brain connectivity and the corresponding graph theory metrics would stabilize after a certain amount of time, requiring different durations of resting-state fNIRS imaging signal acquisition for optimal characterization. In this study, fNIRS data were collected from 18 healthy young subjects who underwent two resting-state scanning runs. For each participant, the hemoglobin signal was preprocessed using independent component analysis (ICA) to reduce physiological noise and other artifacts (e.g., instrumental noise, motion-induced artifacts, and physiological noises) from fNIRS measurement. Finally, we evaluated the influence of fNIRS signal scanning time on the stability and reproducibility of graph theory metrics of brain networks.

## Materials and methods

### Participants and protocol

Twenty-one healthy right-handed subjects (mean age 24.5 years, 17 males and 4 females) participated in this study. Written informed consent was obtained from each subject prior to the experiment. Data collection was carried out according to the protocols approved by the Review Board at the State Key Laboratory of Cognitive Neuroscience and Learning, Beijing Normal University. Resting-state fNIRS data of ~11 min in length from each of two scanning runs (20-min intervals between them) were obtained from each subject. During the scanning, the subjects were asked to relax and remained still with their eyes closed but not to fall asleep. During the interval, the subjects were allowed to open their eyes and move their bodies and heads slightly. The data used in this study was same as in our previous studies that examined graph metrics reliability (Niu et al., 2013) and evaluated brain functional connectivity dynamics (Li et al., 2015).

### Data acquisition

**Figure 1:**
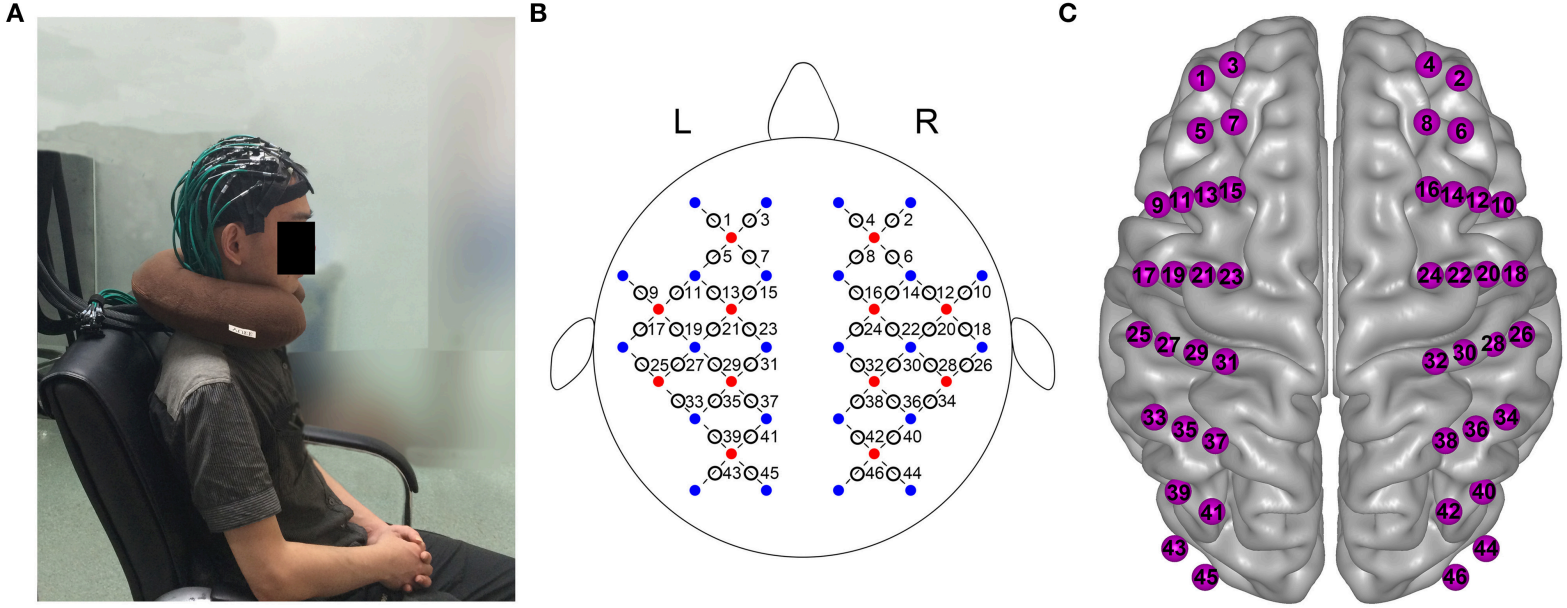
Whole-head fNIRS measurement. **(A)** Photograph of whole-head fNIRS measurement on participant. **(B)** The schematic of whole-head imaging pad (12 sources, red, 24 detectors, blue). The sources and detectors were symmetrically placed on the left and right hemispheres and constituted 46 measurement channels, which allowed for the whole brain (i.e., frontal, temporal, parietal, and occipital lobes) to be measured. **(C)** Anatomical position of each measurement channel.

A continuous wave near-infrared optical imaging system (CW6, TechEn Inc., MA, USA) was used to measure time courses of oxyhemoglobin (HbO) and deoxyhemoglobin (HbR) concentrations at a rate of 25 Hz. The system included 12 laser sources and 24 detectors, with each source including two wavelengths (690 and 830 nm) of near infrared light. The sources and detectors were systematically positioned on the participant's whole head, and the spatial separation between adjacent sources and detectors was set to be 3.2 cm. The configuration resulted in 46 measurement channels that covered the frontal, temporal, parietal, and occipital lobes (Figure 1) of the cerebral cortex. The positions of the probes were consistent with the international 10–20 system of electrode layout.

### Data preprocessing

We used the modified Beer-Lambert law (MBLL) (Cope and Deply, 1988) to compute concentration changes in hemoglobin signals from the attenuation of light through the head at two wavelengths. The time course of hemoglobin concentration was subsequently subjected to a temporal ICA analysis to remove motion-induced artifacts and systematic noise. The resulting data was then band-pass filtered (0.01~0.1 Hz) to obtain low frequency hemodynamic fluctuations (Biswal et al., 1995; White et al., 2009; Sasai et al., 2012). Specifically, the ICA analysis was conducted with the following procedures: extracting steady hemoglobin concentration signals for all participants (e.g., 10 min scanning length in our study), reducing the dimensionality of the hemoglobin data using principal component analysis (PCA) for each participant, conducting ICA analysis on the reduced dimensional data, identifying typical noise components, removing the identified noise from the measured data, and computing “real” neural activity signals. The components related to noise and artifacts were identified from each individual subject based on the following three investigations: temporal profiles, spatial maps, and power spectra. A component would be considered noise if it met one of the following conditions (Zhang et al., 2010): (i) the temporal profile of the component included sudden jumps, slowly varied U or inverted U-shaped spike, or numerous inter-current quick spikes; (ii) the corresponding dominant frequency of the power spectra was outside the range of 0.01~0.1 Hz; (iii) the spatial map of the component showed a global and spatially dispersive pattern. It has been pointed out that the spatial map with global and spatially dispersive pattern could represent systemic interference of superficial layer in the head (Kohon et al., 2007). After identifying these different kinds of noise components, the hemoglobin concentration signal that reflected “real” brain activity was reconstructed by eliminating the components identified as noise from the original hemoglobin time course, by assigning zero in the corresponding column of mixing matrix (Kohon et al., 2007). Finally, we truncated the ICA-based denoising data into 30-s time bins that ranged from 1 to 10 min in order to examine the effect of scanning duration on functional brain connectivity and network metrics. Of note, the procedures of ICA analysis used in here was consistent with our previous studies (Niu et al., 2013; Li et al., 2015) and Zhang et al.'s studies (Zhang et al., 2010, 2011), and it was conducted by using a publicly available software, FastICA v2.5 (http://www.cis.hut.fi/projects/ica/fastica/).

### Functional network connectivity and graph theoretical analysis

#### Functional connectivity (FC) definition

Pearson correlation and cross-correlation are the two most commonly used approaches for measuring inter-regional interactions or functional connectivity (FC) in the fNIRS community. In this study, we simultaneously evaluated the effect of different network construction approaches on the FC and graph metrics stability associated with different fNIRS acquisition durations (i.e., 1~10 min in bins incrementally larger by 30 s). For given time series between any two nodal regions, the Pearson correlation or the cross-correlation was separately calculated to generate a 46 × 46 correlation matrix for each time series and subject. Considering the mean time course for one subject as *X* = (*x*_*i*_(*t*)_*t* = 1, 2, …*N*_), where *x*_*i*_(*t*)_*t* = 1, 2, …*N*_ is the mean time series of the ith region, we calculated these two connectivity metrics as follows:

Pearson's correlation:

(1) $$\text{r}\left({\text{x}}_{\text{i}},{\text{x}}_{\text{j}}\right)=\frac{\sum_{\text{t}=1}^{\text{N}}\left[{\text{x}}_{\text{i}}\left(\text{t}\right)-\bar{{\text{x}}_{\text{i}}}\right]\left[{\text{x}}_{\text{j}}\left(\text{t}\right)-\bar{{\text{x}}_{\text{j}}}\right]}{\surd^{\sum_{\text{t}=1}^{\text{N}}{\left[{\text{x}}_{\text{i}}\left(\text{t}\right)-\bar{{\text{x}}_{\text{i}}}\right]}^{2}}\surd^{\sum_{t=1}^{\text{N}}{\left[{\text{x}}_{\text{j}}\left(t\right)-\bar{{\text{x}}_{\text{j}}}\right]}^{2}}}$$

where $\bar{{\text{x}}_{\text{i}}}$ denotes the average of x_i_.

Cross correlation:

(2) $${\text{r}}_{\text{ij}}(d_{\text{ij}})=\frac{\sum_{\text{t}=1}^{\text{N}}\left[{\text{x}}_{\text{i}}\left(\text{t}\right)-\bar{{\text{x}}_{\text{i}}}\right]\left[{\text{x}}_{\text{j}}\left(\text{t}-{\text{d}}_{\text{ij}}\right)-\bar{{\text{x}}_{\text{j}}}\right]}{\surd^{\sum_{\text{t}=1}^{\text{N}}{\left[{\text{x}}_{\text{i}}\left(\text{t}\right)-\bar{{\text{x}}_{\text{i}}}\right]}^{2}}\surd^{\sum_{\text{t}=1}^{\text{N}}{\left[{\text{x}}_{\text{j}}\left(\text{t}-{\text{d}}_{\text{ij}}\right)-\bar{{\text{x}}_{\text{j}}}\right]}^{2}}}$$

where d_ij_ denotes time delays between the mean time series of the ith and jth regions, and it ranges from 0 to N-1. The maximum r_ij_(d_ij_) in the series of calculation was considered as the functional connectivity strength of these two brain regions.

### Network thresholding

Because there is limited knowledge regarding selection of the network threshold in fNIRS imaging data, we adopted a widely used sparsity threshold, which is also similar to our previous studies (Niu et al., 2012, 2013). Sparsity is defined as the number of existing edges divided by the maximum possible number of edges within a network. The range of the sparsity threshold was chosen from 0.17 to 0.5 (interval = 0.01) considering the small-worldness of human brain networks (Watts and Strogatz, 1998). Thus, for each subject at each time scanning duration, binarized adjacency networks were generated by using these chosen thresholds.

### Network measures

In graph theory, the metrics of network efficiency has been frequently proposed to characterize the capacity of information communication within a network (Latora and Marchiori, 2001, 2003). These related measures have been used to study normal development (Kaustubh et al., 2009; Wu et al., 2013; Cao et al., 2014) and a variety of clinically related brain diseases (Wang et al., 2009; Lynall et al., 2010; Rudie et al., 2012; Yu et al., 2016) because of their conceptual and technical advantages (Achard and Bullmore, 2007; Rubinov and Sporns, 2010). Here, we adopted three typical network efficiency metrics, i.e., nodal efficiency, network local efficiency, and global efficiency, to characterize the ability of information communication in fNIRS brain networks. Specifically, for each subject at each fNIRS signal acquisition duration, the nodal efficiency, network local efficiency, and global efficiency were separately computed by using an in-house FC-NIRS package (Xu et al., 2015) at each sparsity threshold. Furthermore, we also conducted similar calculation on the metrics of network clustering coefficient and nodal betweenness centrality in order to comprehensively examine the effect of fNIRS scanning duration on network metric stability. To exclude the impact of thresholds and to obtain a threshold-independent network evaluation, we further calculated the integral under the curve (AUC) of sparsity threshold values for each network metric (Wang et al., 2011; Niu et al., 2012, 2013) at each time epoch and subject. Specifically, the definitions of these network metrics are summerized as follows:

#### Nodal efficiency

Nodal efficiency (E_nodal_) is a measure that represents the capacity of a node to communicate with the other nodes of the network *G* and is generally defined as follows:

(3) $${\text{E}}_{\text{nodal }}\left(\text{i}\right)=\frac{1}{\text{N}-1}\sum_{\text{i }\neq \text{ j}}\in \text{G}\frac{1}{{\text{d}}_{\text{ij}}}$$

where d_ij_ is the shortest path length between node i and node j, and N is the number of nodes in the network.

#### Nodal betweenness

Nodal betweenness is a measure that characterizes the global role of a node in the brain functional network and is generally defined as follows:

(4) $${\text{b}}_{\text{i}}=\frac{1}{\left(\text{n}-1\right)\left(\text{n}-2\right)}\sum_{\begin{array}{c} \text{h},\text{j}\in \text{N}\\ \text{h}\neq \text{j};\text{h}\neq \text{i},\text{j}\neq \text{i} \end{array}}\frac{\rho_{\text{hj}}\left(\text{i}\right)}{\rho_{\text{hj}}}$$

where ρ_hj_ is the number of shortest paths between h and j, and ρ_hj_ (i)is the number of shortest paths between h and j that pass through i.

#### Network clustering coefficient

Network clustering coefficient is a global measure that characterizes the extent of local interconnectivity and cliquishness of a network and is generally defined as follows:

(5) $$\text{C}=\frac{1}{\text{n}}\sum_{\text{i}\in \text{N}}C_{\text{i}}=\frac{1}{\text{n}}\sum_{\text{i}\in \text{N}}\frac{2{\text{t}}_{\text{i}}}{{\text{k}}_{\text{i}}\left({\text{k}}_{\text{i}}-1\right)}$$

where C_i_ is the clustering coefficient of node i, t_i_ is the actual number of edges between neighbors of node i, and k_i_ is the number of neighbors of node i.

#### Network global efficiency

Global efficiency is a global measure that characterizes information transferring ability in the entire brain network, and it is computed as the mean of nodal efficiency across all nodes of the network (Latora and Marchiori, 2001):

(6) $${\text{E}}_{\text{glob}}\left(\text{G}\right)=\frac{1}{\text{N}\left(\text{N}-1\right)}\sum_{\text{j }\neq \text{ i}\in \text{G}}\frac{1}{{\text{d}}_{\text{ij}}}$$

where d_ij_ is the shortest path length between node i and node j, and N is the number of nodes in the network.

#### Network local efficiency

Network local efficiency represents the efficiency of information flow within the local environment, and it reflects the capability of a network to tolerate faults (Latora and Marchiori, 2001). The local efficiency of network *G* is computed as follows:

(7) $${\text{E}}_{\text{loc}}\left(\text{G}\right)=\frac{1}{\text{N}}\sum_{\text{i}\in \text{G}}{\text{E}}_{\text{glob}}\left({\text{G}}_{\text{i}}\right)$$

where E_glob_(G_i_) the global efficiency of G_i_, the subgraph of the neighbors of node i. The neighbors of node i are defined as the nodes those connect with node i directly.

### Stability evaluation

To evaluate the stability of FC and network efficiency metrics associated with different fNIRS signal acquisition durations, a series of fNIRS data collection durations for FC and graph metric stabilization were contrasted with relatively longer 10-min data. For instance, for FC or the nodal efficiency metric, the linear correlation coefficient was calculated to demonstrate the similarity strength between spatial maps from each short duration data segment and that of the relatively longer 10-min data segments. For local and global efficiency metrics, a statistical analysis (paired *t*-test) was performed to determine the difference between the efficiency values of each short duration data segment and relatively longer 10-min data segments.

### Between-run reproducibility evaluation

We recomputed the FC and network efficiency metrics with the second scan data for all subjects. To assess the between-run reproducibility on the FC and network efficiency measures across different fNIRS scanning durations, we conducted correlation analysis on FC and network efficiency measures between these two runs. Specifically, for the FC pattern, individual FC was first calculated and then transferred into a column vector and conducted correlation analysis for each subject between two runs. The procedure was repeated at each fNIRS signal scanning duration to obtain reproducibility assessment on FC. Similar analysis was conducted in nodal efficiency measure. For network global and local efficiency, the between-run reproducibility on network local and global efficiency was directly measured by calculating Pearson correlation coefficients across all subjects. In addition, we also computed the intra-class correlation coefficient (*ICC ICC* Kong et al., 2007; Niu et al., 2013) for the two-run scanning data at each time duration to examine the effect of different fNIRS scanning durations on the test-retest reliability of FC and network efficiency metrics. The *ICC* is defined as follows:

(8) $$ICC=\frac{MS_{b}-MS_{w}}{MS_{b}+\left(k-1\right)MS_{w}}$$

where *k* is the number of repeated observations per subject, *MS*_*b*_ is the between-subject variance and *MS*_*w*_ is the within-subject variance. Notably, a higher *ICC* value represented a more reliable network measure under the fNIRS scanning duration, whereas a lower *ICC* value represented a less reliable network measure under the fNIRS scanning duration.

### Validation analysis

To validate the reproducibility of our results, we implemented weighted network in addition to the binary network analysis. The detailed description of the weighted network metrics can be found in (Bullmore and Sporns, 2009). The weights of connections in the weighted network in the study survived after thresholding with sparsity from 0.17 to 0.5 with a step of 0.01(similar to binary network analysis) were applied for each time duration bin.

## Results

### Effect of fNIRS scanning duration on FC and network properties stability

**Figure 2:**
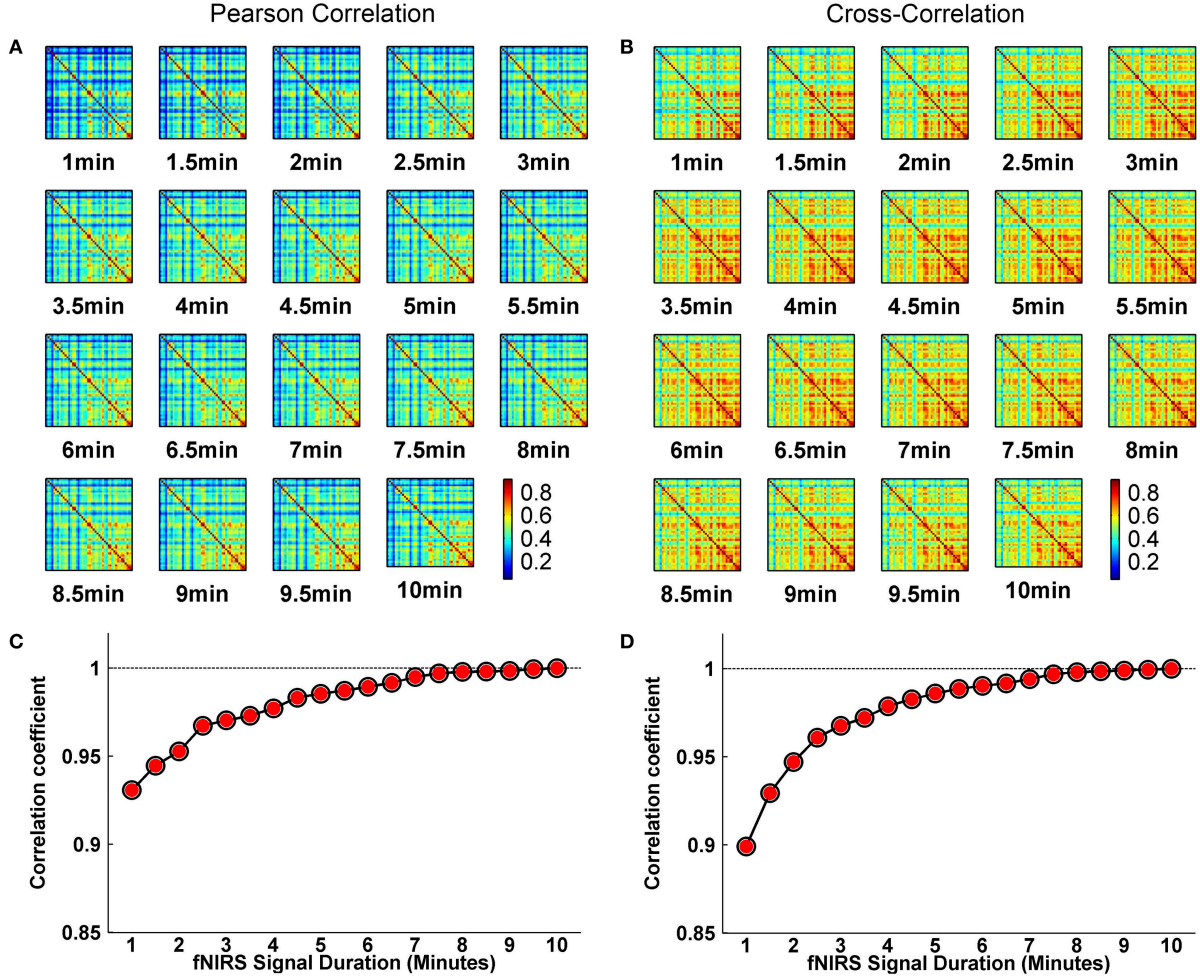
Effect of fNIRS signal acquisition duration on the stability of the spatial FC map. **(A,B)** Graphs show FC patterns plotted vs. the duration of fNIRS signal duration (1.0–10.0 min in 30 s bins). **(C,D)** Between-map correlation coefficients calculated for spatial pattern of FC between short and long (up to 10-min) signal durations. The red-filled circles indicate significant correlation between the 10-min signal duration based spatial FC pattern and spatial FC pattern derived from each fNIRS signal acquisition duration from 1 to 10 min with a 30-s increment. The FC in **(A,C)** and **(B,D)** calculated from Pearson correlation-derived and cross-correlation-derived networks, respectively.

**Figure 3:**
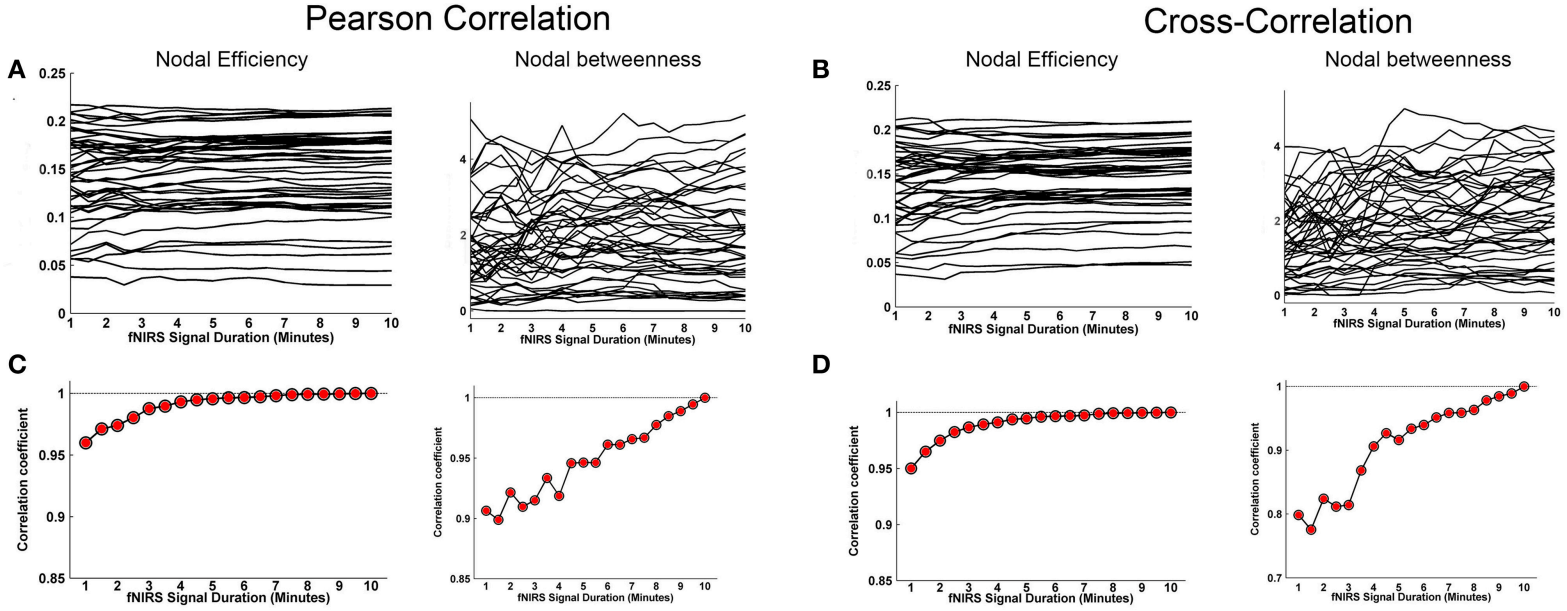
Effect of fNIRS signal acquisition duration on the stability of nodal efficiency and nodal betweenness. The nodal efficiency and nodal betweenness in **(A)** and **(C)** were calculated from Pearson correlation-derived networks. **(A)** Graphs show magnitude of nodal efficiency and nodal betweenness plotted by the duration of fNIRS signal acquisition (1~10 min in bins incrementally larger by 30 s). **(C)** Between-map correlation coefficients calculated between short and long (10-min) signal durations for spatial pattern of nodal efficiency and nodal betweenness, respectively. The red-filled shapes indicate significant correlation between the spatial pattern of nodal efficiency or nodal betweenness associated with a given fNIRS signal acquisition duration and that computed using 10 min of fNIRS data. **(B)** was similar to **(A)** and **(D)** was similar to **(C)** except that the nodal efficiency and nodal betweenness were calculated from cross-correlation-derived networks.

**Figure 4:**
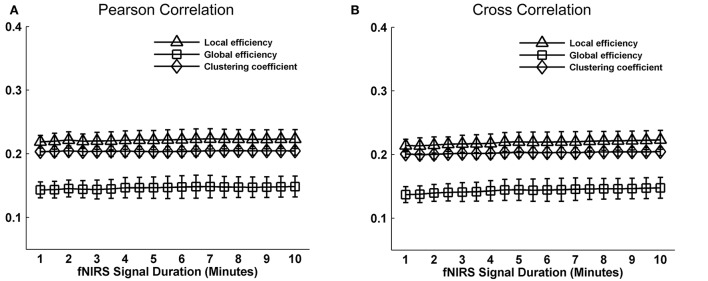
Effect of fNIRS signal acquisition duration on the stability of local efficiency, global efficiency, and clustering coefficient. **(A,B)** Graphs show the magnitude (mean ± *SD*) of local efficiency, global efficiency, and clustering coefficient plotted by duration of fNIRS signal acquisition (1.0~10.0 min in 30-s bins). The efficiency metrics and clustering coefficient in **(A)** and **(B)** calculated from Pearson correlation-derived and cross-correlation-derived networks, respectively. Statistical analysis using paired *t*-test indicates no significant differences in the magnitude of a graph metric associated with a given fNIRS signal acquisition duration compared with the magnitude of the same graph metric when computed by using 10 min of fNIRS data. These data constitute nearly horizontal lines, with little difference between the magnitudes of the computed graph metrics at each data collection duration.

Whole-brain FC and network metrics, i.e., nodal efficiency, nodal betweenness, network local efficiency, global efficiency, and clustering coefficient were separately computed for every subject at each fNIRS signal collection duration. Visually, with increasing scanning durations, the FC maps did not exhibit relatively large pattern variations across both Pearson-correlation (Figure 2A) and cross-correlation (Figure 2B) networks. When contrasted with the relatively longer 10-min data acquisition duration, these results also revealed significant (*p* < 0.001) and strong correlation across each fNIRS signal time bin (the mean correlation coefficients *r* = 0.98 ± 0.03 for Pearson-correlation and *r* = 0.97 ± 0.04 for cross-correlation) (Figures 2C,D). This suggests that the short-time fNIRS signal acquisition duration, e.g., 1 min, can also bring about highly similar FC maps as those calculated from 10-min scanning durations. For nodal efficiency, plots of these efficiency values showed approximately horizontal lines, with small difference between the magnitudes of nodal efficiency across the scanning duration (Figures 3A,B left). For nodal betweenness, plots of these betweenness centrality values showed relatively bigger changes, compared with nodal efficiency plots, between the magnitudes of betweenness values across the scanning duration (Figures 3A,B right). However, the map-map correlation analysis (Figures 3C,D) revealed significant (*p* < 0.001) and strong correlations between short and long scanning duration data for both nodal efficiency and nodal betweenness, indicating almost immediate stability for nodal efficiency and nodal betweenness. This result was consistently found for both Pearson-correlation-derived and cross-correlation-derived nodal efficiency and nodal betweenness. For local efficiency, global efficiency, and clustering coefficient metrics, these plots of the network values also showed similar magnitudes with the increase in scanning duration for the both the Pearson correlation-based network and the cross-correlation-based network (Figures 4A,B). The statistical analysis using paired *t*-tests further revealed no significant difference existed in local or global efficiency metrics between fNIRS signal acquisition duration (*p* > 0.05). This result also demonstrated that the network efficiency and clustering coefficient computed by using the 1.0-min fNIRS signal acquisition duration were no different compared to these measures calculated by using the 10-min fNIRS scanning time.

### Evaluation of between-run reproducibility and reliability

**Figure 5:**
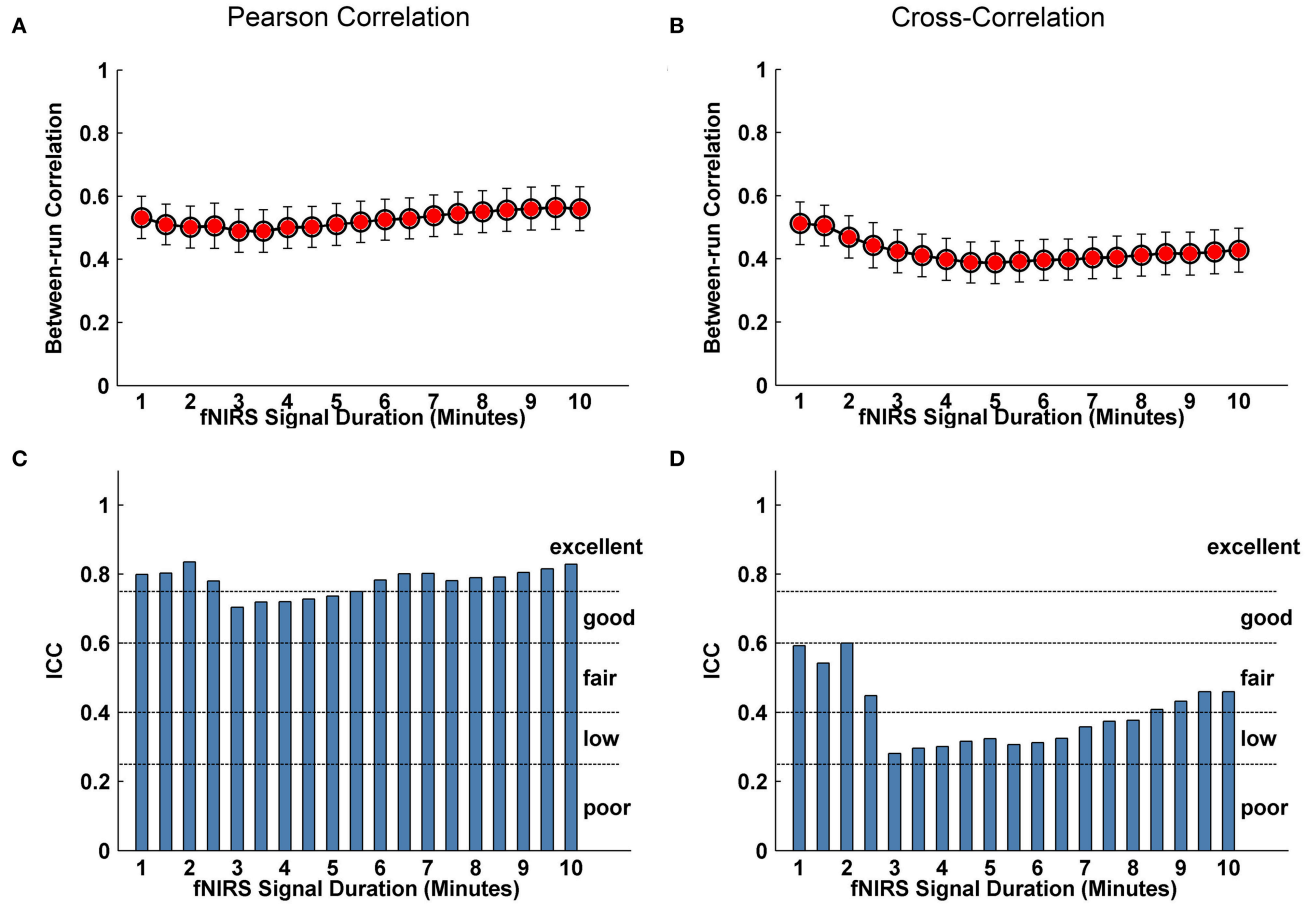
Evaluation of the effect of fNIRS signal acquisition duration on the reproducibility of spatial FC patterns. **(A,B)** Between-run correlation coefficients for FC patterns plotted by duration of fNIRS signal acquisition (1.0~10.0 min in 30-s bins) at individual level. The red-filled circles indicate significant correlations in the spatial patterns of FC map in **(A,C)** and **(B,D)** calculated from Pearson correlation-derived and cross-correlation-derived networks, respectively.

**Figure 6:**
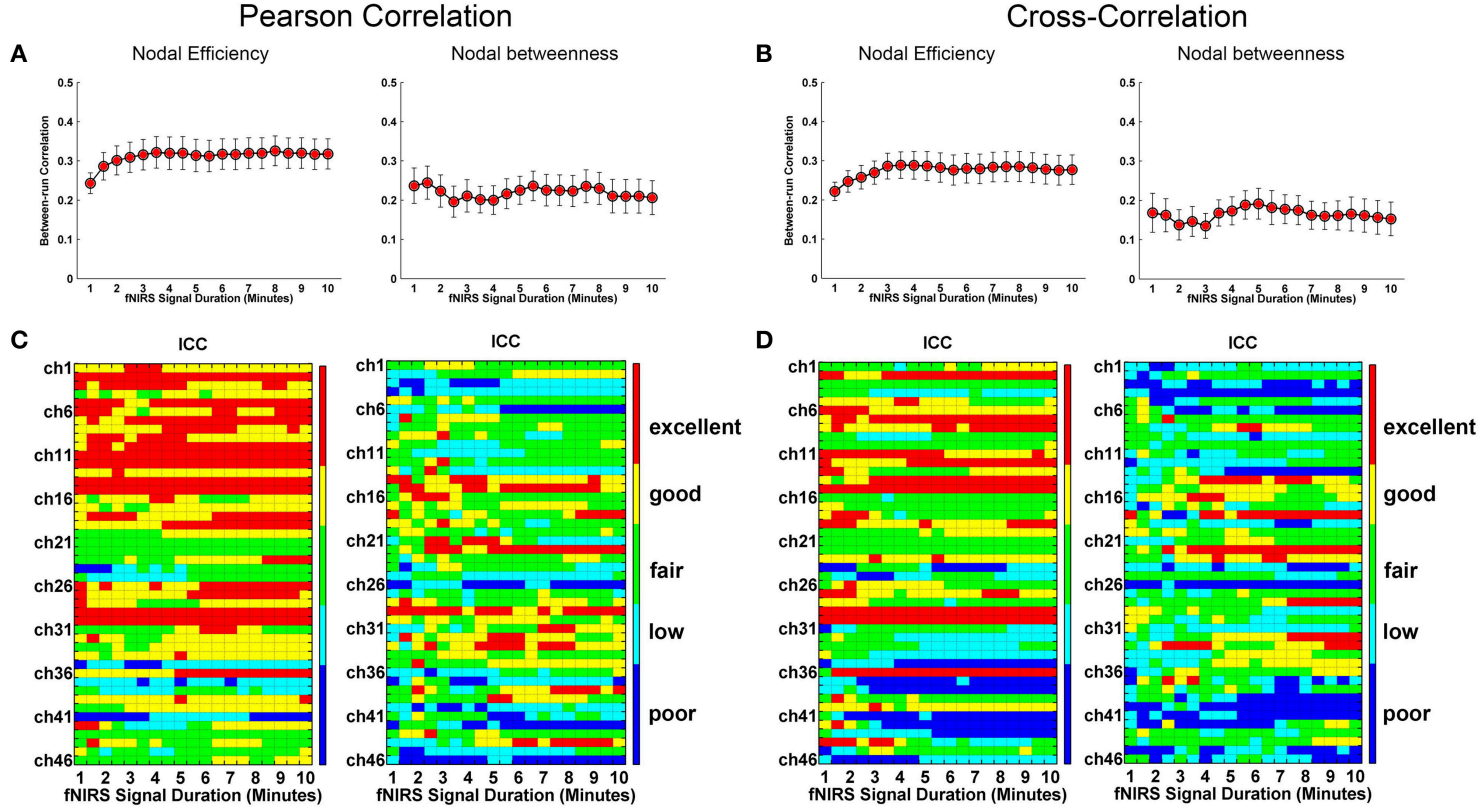
Evaluation of the effect of fNIRS signal acquisition duration on the reproducibility of nodal efficiency and nodal betweenness. The nodal efficiency and nodal betweenness in **(A)** and **(C)** were calculated from Pearson correlation-derived networks. **(A)** Between-run correlation coefficients for nodal efficiency and nodal betweenness plotted by duration of fNIRS signal acquisition (1.0~10.0 min in 30-s bins), respectively. The red-filled shapes indicate significant correlations in the spatial patterns between two runs at the same signal acquisition bin for nodal efficiency and nodal betweenness, respectively. **(C)** The *ICC* values for nodal efficiency and nodal betweenness, respectively. **(B)** was similar to **(A)** and **(D)** was similar to **(C)** except that the nodal efficiency and nodal betweenness were calculated from cross-correlation-derived networks.

**Figure 7:**
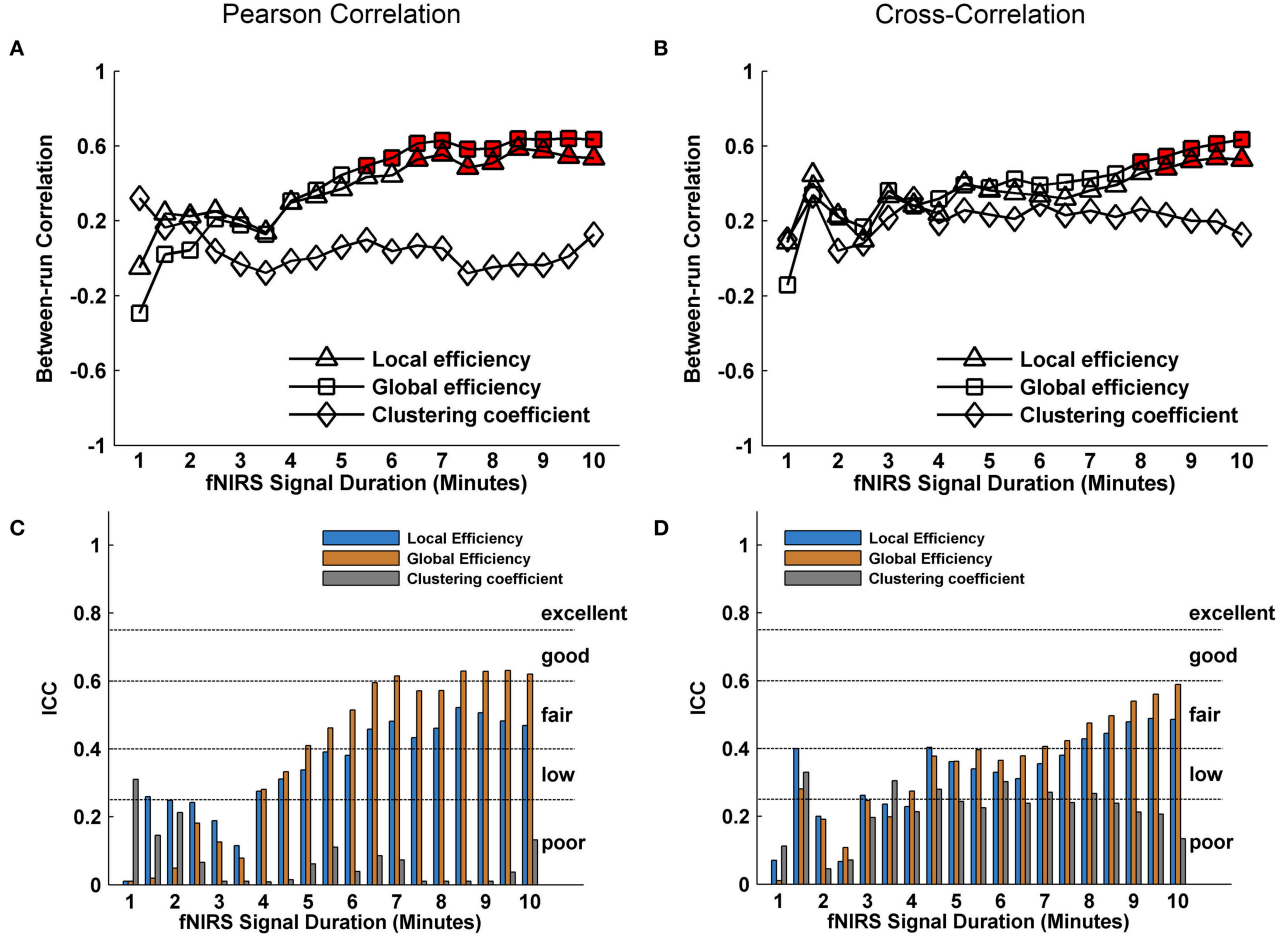
Evaluation of the effect of fNIRS signal acquisition duration on the reproducibility of local efficiency, global efficiency and clustering coefficient. The local efficiency, global efficiency, and clustering coefficient in **(A)** and **(C)** were calculated from Pearson correlation-derived networks. **(A)** Between-run correlation coefficients for the global network metrics plotted by duration of fNIRS signal acquisition (1.0–10.0 min in 30-s bins). The red-filled shapes indicate significant correlations between two runs at the same signal acquisition bin for the global network measures. **(C)** The *ICC* values for the global network metrics, respectively. **(B)** was similar to **(A)** and **(D)** was similar to **(C)** except that the local efficiency, global efficiency, and clustering coefficient were calculated from cross-correlation-derived networks.

We further evaluated the effect of resting-state fNIRS signal acquisition duration on the reproducibility and reliability of FC and these network metrics mentioned above. For the Pearson-correlation network, the FC maps showed high similarity between the two runs at each fNIRS signal time bin (mean correlation coefficients *r* = 0.53 ± 0.067, *p* < 0.001) (Figure 5A), and the *ICC* values of the FC also demonstrated approximately or equally excellent reliability as the scanning duration ranging from 1 to 10 min (Figure 5C). Similarly, the nodal efficiency and nodal betweenness also showed good reproducibility between two scanning runs (*r* = 0.31 ± 0.04 for nodal efficiency, and *r* = 0.28 ± 0.04 for nodal betweenness, *p* < 0.001) (Figure 6A) and high reliability (mainly for fair to excellent levels) (Figure 6C) with the increase in the scanning duration. This suggested that fNIRS data as short as a 1-min resting-state fNIRS signal can yield reproducible and reliable FC maps, nodal efficiencies and nodal betweenness. For global network metrics, a high repeatability between runs was found for global and local efficiency as scanning durations ≥ 5 min (Figure 7A); correspondingly, the analysis of *ICC* values also revealed better reliability for relatively longer scanning durations, e.g., longer than 5 min (Figure 7C). However, the clustering coefficient showed relatively lower between-run repeatability and reliability.

For cross-correlation constructed networks, good repeatability between runs is found in FC maps (Figure 5B, mean correlation coefficients *r* = 0.43 ± 0.056, *p* < 0.001), nodal efficiency (Figure 6B, *r* = 0.22 ± 0.04, *p* < 0.001), and nodal betweenness (*r* = 0.16 ± 0.04, *p* < 0.001) for each time bin, respectively. However, the reliability for FC and nodal efficiency is much lower compared to that from the Pearson-correlation network, e.g., the general reliabilities for the FC (Figure 5D) and nodal efficiency (Figure 6D) were low and fair levels, respectively. For global and local efficiency, a good repeatability between runs was found for scanning duration ≥ 8 min (Figure 7B) and a high reliability was found for scanning duration ≥5 min (Figure 7D). Similarly, the clustering coefficient calculated using cross-correlation constructed networks showed relatively low between-run repeatability and reliability.

### Validation results

All the network analysis results using weighted network are presented in Supplementary Figures 1–4 in Supplementary Material. In general, we found few differences in the main results using weighted network analysis when compared to those using binary network analysis. For example, nodal efficiency and nodal betweenness stabilized and were reproducible after 1 min of fNIRS signal acquisition, whereas network local efficiency, global efficiency, and clustering coefficient stabilized after 1 min and were reproducible after 5 min of fNIRS signal acquisition only for network local and global efficiency.

## Discussion

For functional network connectivity methods to be useful in practical applications, non-invasive fNIRS brain imaging techniques need provide direct evidence to confirm that sufficient fNIRS imaging data with shortest reasonable scanning time was used for data analysis, which—to our knowledge—has not been investigated for FC and graph theoretical study. Here, our results showed that fNIRS brain FC and network properties (e.g., nodal efficiency, nodal betweenness, network local, and global efficiency) can be accurately calculated from as short as 1 min of resting-state fNIRS imaging data. A series of fNIRS data with different collection durations for graph metric stabilization is contrasted with data recorded with relatively longer durations of 10 min, and the results revealed high similarity in the FC and graph theory metrics between short and long acquisition durations. These results are also consistent with our hypothesis that the correlation coefficient data and the corresponding computed network measures stabilize with increasing scanning time, and such results indicate that different resting-state fNIRS imaging durations might be applied depending on the outcome of interest. Although these data were consistent with our proposed hypothesis, the magnitude in fNIRS signal collection duration required for data reproducibility between the metrics was somewhat different. For example, the FC, nodal efficiency and nodal betweenness metrics could be reliably reproduced between runs after 1-min fNIRS signal acquisition duration, whereas the local and global efficiency could be reliably reproduced after ~5-min fNIRS signal acquisition durations. One possible explanation for the quick reproducibility of both the FC and nodal centrality is that the intrinsic organizational configuration between nodal brain regions emerged at the earliest scanning time (Whitlow et al., 2011). However, for local and global efficiency, the metric reproducibility depends on the elaborate structure of the measured networks, whereas the elaborate structure might be sensitive to the scanning duration of the fNIRS signal. Furthermore, it is possible that the local and global efficiency metrics characterize aspects of intrinsic network properties in the brain, while the intrinsic network properties can be less robustly calculated using short fNIRS signal(s) (Wang et al., 2011; Niu et al., 2013). This was also found in the network clustering coefficient (Figure 7). In the future, studies based on large sample data sets are expected to provide further evidence to understand the temporal dynamics underlying the differences in network reproducibility/reliability.

Notably, the current study suggests that short data, i.e., 1 min of fNIRS signal acquisition is sufficient for obtaining FC and network metric stabilization, and is inconsistent with previous fMRI reports. Using fMRI, Van Dijk et al. demonstrated ~5–6 min (Van Dijk et al., 2010) and Whitlow reported 2 min (Whitlow et al., 2011) of minimum data collection duration were required for FC stability. Recent studies from Laumann et al. (2015), Tomasi et al. (2016), Birn et al. (2013), and Dawson et al. (2013) showed that longer data collection duration (e.g., >9 min) is required to yield the stable FC pattern. The fNIRS data owns a high temporal sampling rate (e.g., 25 Hz for the current study) for recording the dynamic hemodynamic signals within the brain, which is a marked advantage and is also a difference between fNIRS data and fMRI data. Therefore, it is unknown whether the sampling rate difference leads to the discrepancy in the observed results. Beyond the technical factors, the discrepancies among the results could be attributed to the analysis methods (including correlation analysis and variability analysis), the network size (46 and 116), and the network threshold selection (cost from 0.1 to 0.4 and sparsity from 0.17 to 0.5) between previous studies and the current study. Resting-state imaging data capture the information of complex integration among various brain regions and that the integration always exhibits a dynamic, time-varying fashion on the order of seconds or minutes (Bullmore and Sporns, 2009; Chu et al., 2012; Niu et al., 2013). Therefore, it would be of great interest to compare FC and graphic metric stabilization using the simultaneous acquisition of fNIRS and BOLD-fMRI in the future.

We evaluated the effect of different network construction approaches (Pearson correlation and cross-correlation) on the FC and graph metrics stability associated with different fNIRS acquisition durations. Although our main aim was not to determine which type of network construction approach is optimal for obtaining the minimum scanning stability and reproducibility of FC and network metrics, we did find that the reliability of the FC, the nodal efficiency and betweenness metric, and the run-run repeatability for local and global efficiencies the clustering coefficients were much better when employing the Pearson correlation approach. This conclusion is also compatible with a previous fMRI study that demonstrated graph metrics derived from Pearson's-correlation-based networks are more reliable for both short-term scans and long-term scans (Liang et al., 2012). However, when considering only the stability of FC and graph theory metrics associated with different fNIRS imaging durations, the two correlation approaches are not significantly different. As such, a 1 min duration seems to be an appropriate choice for brain imaging studies in which graph theoretical analyses of functional brain networks are used.

The systemic artifacts from the scalp and the skull is considered as a dominant noise sources in resting-state fNIRS signal, and has been an active area of research in recent years. Currently, there are three different types of methods developed for separating the noise sources from real neuronal signals, which includes short separation regression (Gagnon et al., 2011; Saager et al., 2011), ICA (Kohon et al., 2007), and adaptive filter (Zhang et al., 2007, 2009). These noise reduction approaches are playing important roles in improving signal quality of spontaneous neural activity. Among them, both the short-distance regression and the adaptive filter methods require a specially designed measurement channels (about 1 cm in spatial separation between source and detector) to record the superficial signal from the scalp and the skull. More importantly, this design needs to be considered in advance and simultaneously accomplished with the measurement in the regions of interest during the stage of data collection. Recently, Gagnon et al. pointed out that the location of short separation measurement placed in participants' head seriously impacted the performance of superficial noise regression in resting-state fNIRS signal (Gagnon et al., 2012). As such, considering the number and location of short-separation channels is of great importance and essential while applying these two methods. In contrast, the ICA is a data-driven method and does not require prior considerations for short-channel measurement during experimental acquisition. Due to the ability of blind source separation, ICA can separate multiple types of noise and artifacts from the measured data. This has been empirically confirmed in both task-based and resting-state fMRI studies. With the ICA method, Zhang et al. identified and removed several noise components in the resting-state fNIRS signal including head motion noise and physiological artifacts. They also confirmed the superior performance for ICA-based noise reduction approach in identifying functional connectivity (Zhang et al., 2010, 2011). Similarly, in our previous studies (Niu et al., 2013; Li et al., 2015), we also demonstrated the usefulness of ICA approach on removing typical noise components by evaluating the between-run reproducibility and reliability for graph metrics and brain functional connectivity dynamics. Nevertheless, it is also noted that the characteristic of blind source separation of the ICA approach is not based on physiological components and could lead to difficulties to completely identify and remove physiological and neural noise components from the measured signals. Future studies are needed to further test whether the current results could be remained as utilizing recording respiration and heart rates while collecting resting-state fNIRS data or other noise-reduction approaches simultaneously (e.g., short-separation measurement).

A few issues need to be further addressed. First, while we adopted different network construction approaches (Pearson's correlation and cross-correlation) to obtain graph metrics of the brain network, we aim not to provide a gold standard for the selection of network construction approaches but to primarily observe how the correlation approaches affect the reproducibility of graph metrics along with different temporal trajectories. Certainly, the network properties and their reproducibility can also be affected by some other technical details, for example, whether there exists global signal regression and how to select the frequency band during preprocessing, which is important to examine but beyond the scope of this paper and is worth conducting separate studies in the future. Second, we only evaluated several typical graph theory metrics associated with increasing fNIRS signal acquisition durations, and thus, it remains unknown whether the current findings, i.e., that FC and network efficiency metrics can be accurately calculated from as little as 1.0 min of fNIRS scanning duration, are valid for the other network metrics. Third, we observed temporal stability of graph metrics based on an integrated sparsity threshold, but it is also interesting to investigate the influence of distinct network sparsity thresholds on the reproducibility of graph metrics computed from different fNIRS acquisition durations. Finally, the resting-state fNIRS imaging data used in this study was from healthy adult participants. As such, the current results have not been validated in the other participant population (e.g., early children and clinical populations). It is known that the developmental aspects of the children brains and abnormalities of the central nervous system in patients may change the stability of the FC and graph theory metrics, possibly requiring longer fNIRS data collection durations. Therefore, it is important to explore possible specificities of such populations with respect to specific fNIRS imaging durations, which may have important implications in the application of network analyses to healthy and diseased brains.

In summary, as little as 1 min of resting-state fNIRS imaging signal may be sufficient to obtain stable graph theory metrics for brain network study. Thus, our finding provides direct evidence for healthy adult studies involving to the choice of the resting-state fNIRS scanning duration in functional brain connectivity and topological metric stability of brain connectivity network.

## Author contributions

SG, XL, and HN put forward academic problem; SG analyzed the data and wrote part of the manuscript; HN and BB revised the manuscript.

### Conflict of interest statement

The authors declare that the research was conducted in the absence of any commercial or financial relationships that could be construed as a potential conflict of interest.
