# Supplementary material for: Effect of Resting-State fNIRS Scanning Duration on Functional Brain Connectivity and Graph Theory Metrics of Brain Network
Source: Front Neurosci. 2017 Jul 20;11:392. doi: 10.3389/fnins.2017.00392 (PMC5517460; doi:10.3389/fnins.2017.00392)
Supplement: Supplementary file 1 [file Image1.PDF]

Supplementary Figures

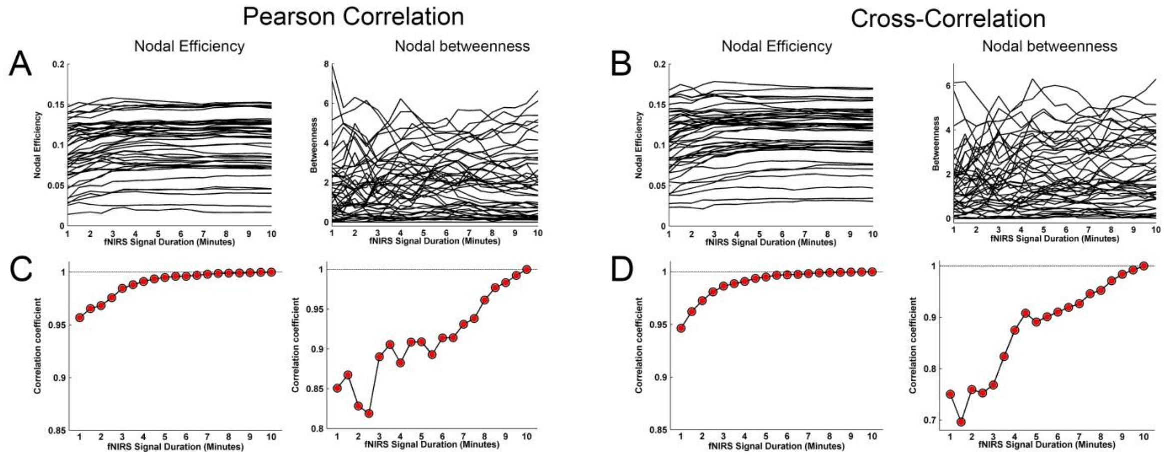

Supplementary Figure 1. Effect of fNIRS signal acquisition duration on the stability of nodal efficiency and nodal betweenness. The nodal centrality metrics were calculated from weighted brain networks. The nodal efficiency and nodal betweenness in (A) and (C) were calculated from Pearson correlation-derived networks. (A) Graphs show magnitude of nodal efficiency and nodal betweenness plotted by the duration of fNIRS signal acquisition (1~10 minutes in bins incrementally larger by 30 seconds). (C) Between-map correlation coefficients calculated between short and long (10-minute) signal durations for spatial pattern of nodal efficiency and nodal betweenness, respectively. The red-filled shapes indicate significant correlation between the spatial pattern of nodal efficiency or nodal betweenness associated with a given fNIRS signal acquisition duration and that computed using 10 minutes of fNIRS data. (B) was similar to (A) and (D) was similar to(C) except that the nodal efficiency and nodal betweenness were calculated from cross-correlation-derived networks.

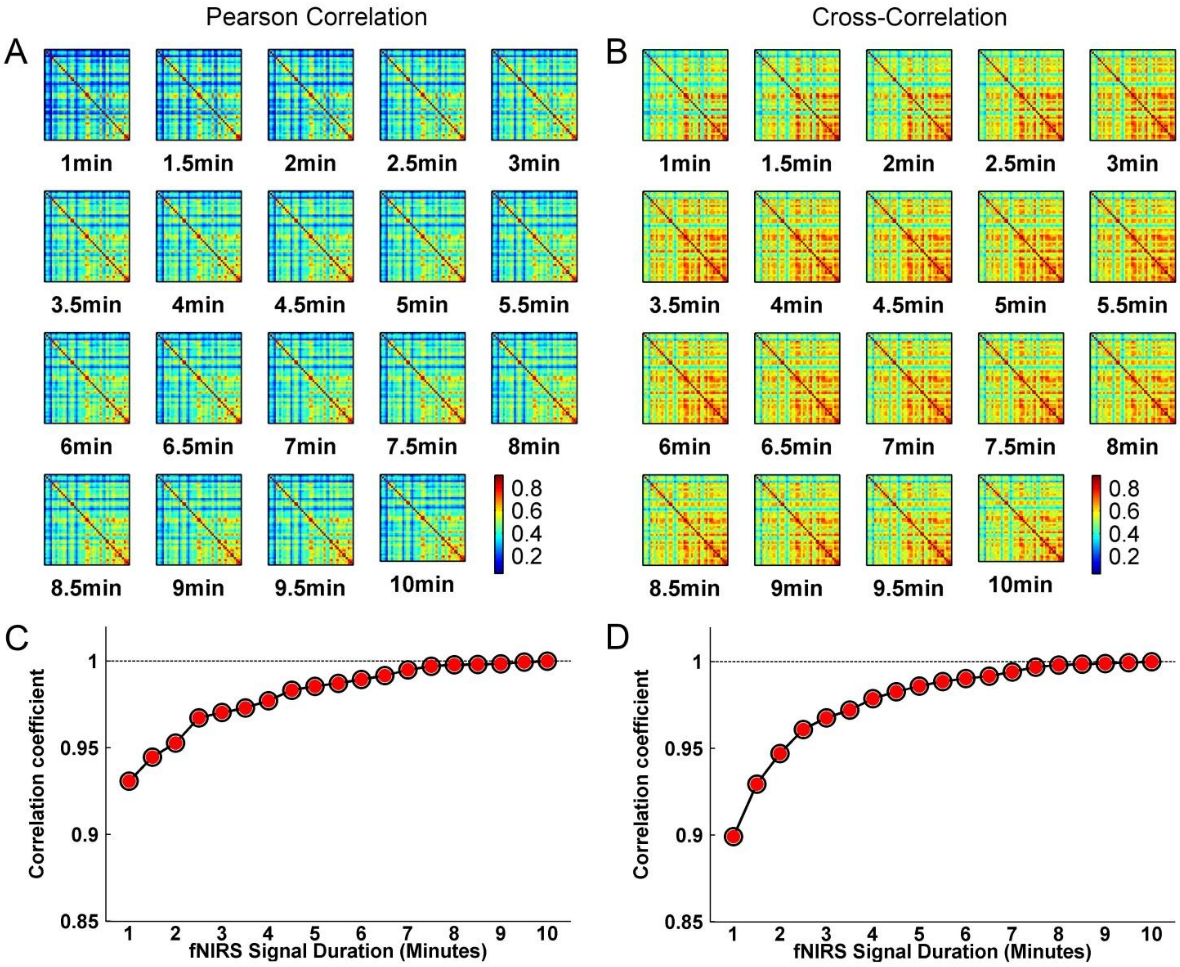

Supplementary Figure 2. Effect of fNIRS signal acquisition duration on the stability of local efficiency, global efficiency and clustering coefficient. The global network metrics were calculated from weighted brain networks. (A-B) Graphs show the magnitude (mean±SD) of local efficiency, global efficiency and clustering coefficient plotted by duration of fNIRS signal acquisition (1.0-10.0 minutes in 30-sec bins). The efficiency metrics and clustering coefficient in (A) and (B) calculated from Pearson correlation-derived and cross-correlation-derived networks, respectively. Statistical analysis using paired t-test indicates no significant differences in the magnitude of a graph metric associated with a given fNIRS signal acquisition duration compared with the magnitude of the same graph metric when computed by using 10 minutes of fNIRS data. These data constitute nearly horizontal lines, with little difference between the magnitudes of the computed graph metrics at each data collection duration.

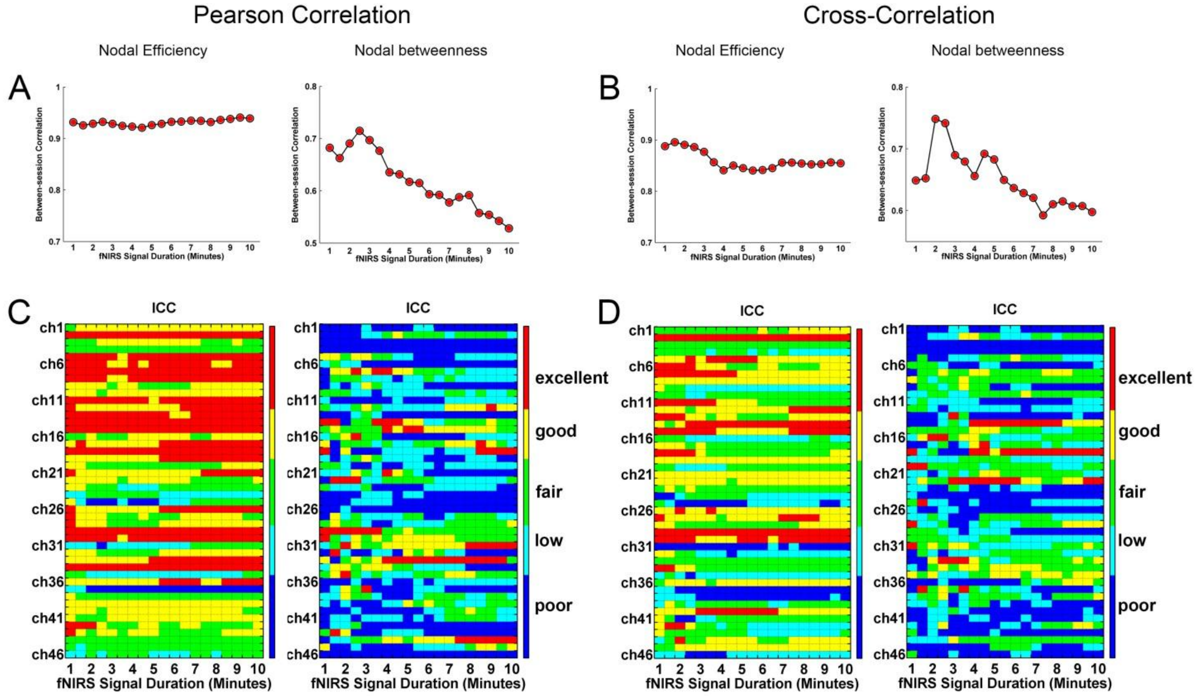

Supplementary Figure 3. Evaluation of fNIRS signal acquisition duration on the reproducibility of nodal efficiency and nodal betweenness. The nodal centrality metrics were calculated from weighted brain networks. The nodal efficiency and nodal betweenness in (A) and (C) were calculated from Pearson correlation-derived networks. (A) Between-session correlation coefficients for nodal efficiency and nodal betweenness plotted by duration of fNIRS signal acquisition (1.0-10.0 minutes in 30-sec bins), respectively. The red-filled shapes indicate significant correlations in the spatial patterns between two sessions at the same signal acquisition bin for nodal efficiency and nodal betweenness, respectively. (C) The ICC values for nodal efficiency and nodal betweenness, respectively. (B) was similar to (A) and (D) was similar to(C) except that the nodal efficiency and nodal betweenness were calculated from cross-correlation-derived networks.

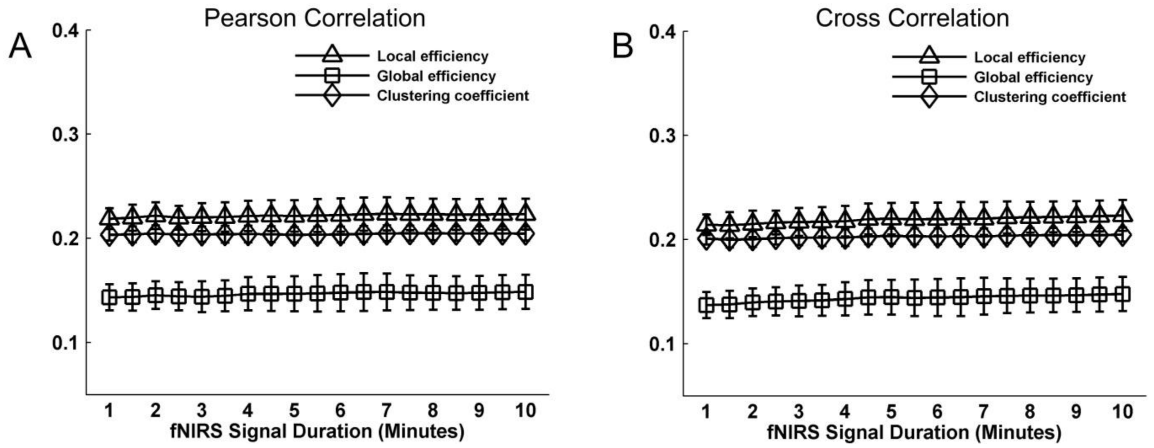

Supplementary Figure 4. Evaluation of fNIRS signal acquisition duration on the reproducibility of local efficiency, global efficiency and clustering coefficient. The global network metrics were calculated from weighted brain networks. The local efficiency, global efficiency and clustering coefficient in (A) and (C) were calculated from Pearson correlation-derived networks. (A) Between-session correlation coefficients for the global network metrics plotted by duration of fNIRS signal acquisition (1.0-10.0 minutes in 30-sec bins). The red-filled shapes indicate significant correlations between two sessions at the same signal acquisition bin for the global network measures. (C) The ICC values for the global network metrics, respectively. (B) was similar to (A) and (D) was similar to(C) except that the local efficiency, global efficiency and clustering coefficient were calculated from cross-correlation-derived networks.
